# Hippocampal-Prefrontal Circuit and Disrupted Functional Connectivity in Psychiatric and Neurodegenerative Disorders

**DOI:** 10.1155/2015/810548

**Published:** 2015-04-01

**Authors:** Ming Li, Cheng Long, Li Yang

**Affiliations:** ^1^School of Psychology, South China Normal University, Guangzhou 510631, China; ^2^School of Life Sciences, South China Normal University, Guangzhou 510631, China

## Abstract

In rodents, the hippocampus has been studied extensively as part of a brain system responsible for learning and memory, and the prefrontal cortex (PFC) participates in numerous cognitive functions including working memory, flexibility, decision making, and rewarding learning. The neuronal projections from the hippocampus, either directly or indirectly, to the PFC, referred to as the hippocampal-prefrontal cortex (Hip-PFC) circuit, play a critical role in cognitive and emotional regulation and memory consolidation. Although in certain psychiatric and neurodegenerative diseases, structural connectivity viewed by imaging techniques has been consistently found to be associated with clinical phenotype and disease severity, the focus has moved towards the investigation of connectivity correlates of molecular pathology and coupling of oscillation. Moreover, functional and structural connectivity measures have been emerging as potential intermediate biomarkers for neuronal disorders. In this review, we summarize progress on the anatomic, molecular, and electrophysiological characters of the Hip-PFC circuit in cognition and emotion processes with an emphasis on oscillation and functional connectivity, revealing a disrupted Hip-PFC connectivity and electrical activity in psychiatric and neurodegenerative disorders as a promising candidate of neural marker for neuronal disorders.

## 1. Introduction

The Hip-PFC pathway comprises the major efferent anatomical connection from hippocampal formation, through monosynaptic and/or polysynaptic projections, to PFC, implicating a critical role of the Hip-PFC circuit in the anatomical and functional coupling of the two regions. Interactions between the hippocampus and the PFC are of major interest in understanding the neurobiology of psychiatric and neurodegenerative disorders. There are both direct- and indirect-Hip-PFC pathways. The direct-Hip-PFC pathway manifests the remarkable monosynaptic unidirectional projection between these two areas [1] and plays vital roles in cognitive processing [2]. In Hip-PFC circuit, a symptom is hardly ever exclusive to a particular psychiatric or a particular neurodegenerative disorder. For instance, cognitive impairment, associated with abnormal hippocampal and PFC function, is a primary cause of disability in schizophrenia, but it also emerges in depression [3, 4]. Although psychiatric and neurodegenerative disorders each produce complex pathophysiology, the similarity in symptoms reveals the outcome of disruption in a shared brain circuit. To understand the deficits in cognition and emotional regulation that leads to these disorders, it is important to reveal the property, function, and connectivity between the PFC and hippocampus.

## 2. Anatomical Connectivity between the Hippocampus and PFC

### 2.1. Direct-Hip-PFC Projection

The direct-Hip-PFC pathway originates from the CA1 region of hippocampus and subiculum [5, 6]. In rats, neurons in the ventral CA1 and subiculum are selectively projecting to prelimbic medial prefrontal cortex (mPFC) and orbitomedial frontal cortex [7, 8]. In comparison, the dorsal CA1 projects lightly to the retrosplenial area of the cingulate region [9]. The fibers navigate ipsilateral PFC through the fimbria/fornix system before terminating in the infralimbic (IL) and the prelimbic (PL) PFC and anterior cingulate cortex [8–10]. Single pulse stimulation in the hippocampus evoked early excitatory postsynaptic potentials (EPSPs) and later inhibitory postsynaptic potentials (IPSCs) in pyramidal cells of PFC [11]. In rodents, immune cytology study shows that the hippocampal efferent innervates the PFC excitatory pyramidal cells and GABAergic interneurons in a monosynaptic manner [12, 13]. Though fine details of the Hip-PFC connectivity in humans are still missing due to lack of intact powerful tract-tracing techniques, diffusion-weighted imaging (DWI) measurements demonstrate that the direct projection originating from hippocampus to PFC is similar in both human and monkeys [14], indicating that data obtained from other primates in this regard may resemble the situation in humans. Furthermore, hippocampal lesions generate disruptions both in fimbria/fornix and in white matter integrity of ventral mPFC in monkeys [15], referring to the Hip-PFC pathway as the major projection originating in the hippocampus that directly innervates the PFC in primates, as well as in rodents.

### 2.2. Hip-PFC Loops

In addition to monosynaptic Hip-PFC projections, there are complex multisynaptic routes between the hippocampus and PFC. We focus here on those which have been shown to be critically involved in higher cognitive function and several major brain disorders.

#### 2.2.1. Hip-NAc-VTA-PFC Loop

Though hippocampus receives direct projection from the ventral tegmental area (VTA), it innervates the VTA through a multisynaptic pathway involving the glutamatergic efferent innervating nucleus accumbens (NAc) [16, 17]. To be precise, the ventral hippocampus (vHip) glutamatergic neurons project to the shell region of NAc, whereas the dorsal hippocampus sends glutamatergic projections to the core region of NAc [18]. The NAc sends GABAergic inhibitory projections to dopaminergic neurons of the VTA [19, 20]. VTA has reciprocal projections with many forebrain structures including the NAc, dorsal striatum, amygdala, and mPFC [21]. Two major subdivisions of the mPFC, IL and the PL, send direct projections to the VTA [22]. The hippocampal neurons receive midbrain dopamine projections originating from the VTA [16, 17]. Activation of the VTA results in dopamine release in the hippocampus which is important for consolidating long-term potentiation (LTP) in CA1 [23]. Thus, the hippocampus and VTA dopaminergic loop may affect hippocampal-dependent learning and regulate the flow of hippocampal short-term memory into the PFC long-term memory [16, 17].

#### 2.2.2. The vHip-BLA-PFC Loop

Within the vHip, neuroanatomical studies have shown that the ventral CA1 and the ventral subiculum project to not only the mPFC, but also the basolateral amygdala (BLA) [24]. Although the majority of ventral CA1 and ventral subiculum neurons send projection to either BLA or mPFC, some vHip neurons project to both areas (dual-projecting neurons) [25]. These dual-projecting hippocampal neurons may be particularly crucial for coordinating mPFC and BLA activity during memory retrieval, a function suggested by similar consequences of vHip-PL and vHip-BLA disconnections on fear renewal [26]. Meanwhile, optogenetic and pharmacological study in mice showed that BLA inputs to the vHip modulate social behaviors in a bidirectional manner [27] indicating a reciprocal connectivity between these two structures.

Excitatory inputs from BLA densely arborize within superficial layers of mPFC [28, 29] and form synapses with layer II pyramidal neurons [30]. Two distinct pyramidal cell populations within PL layer II, which project either to the contralateral mPFC or to the BLA, have been identified by optogenetics [30]. PL-BLA inputs which target spines near the soma of BLA neurons elicit stronger EPSCs than projections targeting the dendrite [30]. Disruption of the PFC-BLA pathway increased choice for large, risky rewards [31]. This unique interconnectivity between the PL and BLA may enable highly efficient reciprocal interaction, which could be crucial for top-down control of emotion [32] and fear and anxiety [33].

#### 2.2.3. Hip-Thalamus-PFC Loop

The hippocampus and anterior thalamic nuclei form part of an interconnected network involved in memory formation which is the central pathway comprising the direct projections from the hippocampus to the anterior thalamic nuclei. Additional pathways involve the internal capsule, associating the dorsal subiculum with the anteromedial thalamic nucleus, as well as the postsubiculum to the anterodorsal thalamic nuclei in rat [34]. The PFC receives projections from several other thalamic nuclei, including the mediodorsal, the anterior medial, the ventral anterior, the medial pulvinar, and midline and intralaminar nuclei [35, 36]. The anterior medial nucleus receives robust projections from the hippocampus [37]. It is worth noting that thalamic and hippocampal inputs to the mPFC show different distribution: the ventral CA1 targets primarily the deep layers of PFC [8], and the mediodorsal nucleus of the thalamus, which is the primary thalamic afferent of the mPFC, mostly projects to layer III [38, 39]. PFC is meanwhile regulated by an ascending activating system involving projections to layer I from those nonspecific thalamocortical relay neurons located predominantly in the paralaminar, intralaminar, and midline thalamic nuclei [40, 41]. The thalamocortical circuits that carry rapid signals over sustained periods in layer I of PFC may contribute to the celebrated propensity of this cortical area to process attention and memory associated with high frequency oscillations and persistent activity [42, 43].

It has been proposed that the reciprocal projections between PFC and thalamic nuclei may initiate brain wave oscillations, associated with consciousness, attention, and executive control [44, 45].

### 2.3. The PFC Efferent and PFC-RE-Hip Loop

**Figure 1:**
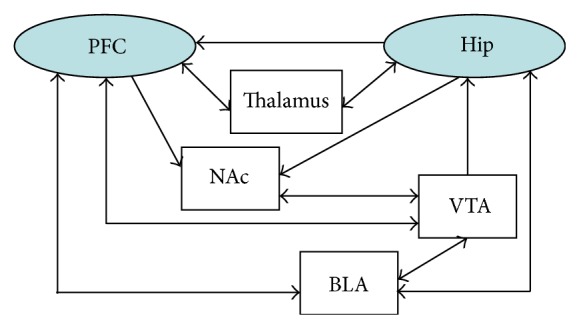
Diagram shows the Hip-PFC pathway. The Hip-PFC pathway involves monosynaptic projection and multisynaptic connection (PFC: prefrontal cortex, Hip: hippocampus, RE: nucleus reuniens, NAc: nucleus accumbens, BLA: basolateral amygdala, and VTA: ventral tegmental area).

Though there is no direct-Hip-PFC projection, hippocampal activity can be affected by PFC via various relay structures. The nucleus reuniens (RE) is the main nucleus of thalamus that receives mPFC innervation and transmits processed information to the hippocampus by monosynaptic projection (Figure 1) [46]. Particularly, the ventral mPFC exclusively targets midline/medial structures of the thalamus. While dorsal mPFC distributes primarily to the intralaminar, ventral, and lateral thalamus. RE fibers form excitatory contacts predominantly on distal dendrites of pyramidal cells in stratum lacunosum-moleculare of CA1 [47], forming a circuit as hippocampus-ventral mPFC-RE-hippocampus.

## 3. Synaptic Response and Plasticity of Hip-PFC Projections

Both pyramidal and local GABAergic neurons in the mPFC are directly targeted by the thalamic [48] and hippocampal glutamatergic afferents [11, 13], such that thalamic as well as hippocampal inputs provoke EPSPs and/or IPSPs sequence in neocortical pyramidal neurons [11, 49]. Short-latency AMPA-receptor mediated excitation in the PL mPFC by electrical stimulation of vHip [50] is followed by inhibition caused by monosynaptic excitation of GABAergic interneurons [11], contributing to feed-forward inhibition of these pyramidal neurons [13].

The Hip-PFC pathway demonstrates synaptic plasticity. Repetitive electrical stimulation in the ventral subiculum or CA1 induces LTP or long-term depression (LTD) in the PFC [51–53] and a short-term synaptic plasticity as well. High-frequency stimulation of BLA, which has reciprocal connections with both vHip and mPFC, prevents the subsequent induction of LTP in Hip-PFC pathway [54]. Consider the requirement of Hip-PFC pathway in transporting hippocampal memory to cortex in behavioral study [55]; these data suggest that the Hip-PFC synaptic plasticity may resemble memory consolidation process [56].

## 4. Working Memory and the Hip-PFC Pathway

Working memory is a task that is used to evaluate the transient holding and processing of new and already stored information, an important process for reasoning, comprehension, learning, and memory updating. Rodent experiments involving asymmetric pathway disconnection methods have shown that both PFC and hippocampus functionally interact during working memory [55] especially in situations with increased task demand [57]. Hippocampus inputs to PL/IL provide an essential projection by which spatial information can be integrated into the cognitive process mediated by the PFC [55]. Lesions of PL/IL induce deficits in the short-term maintenance of information, including delayed alternation and delayed response tasks [58–60]. During working memory performance, neuronal firing and coordinated network activity are observed in PFC and hippocampus in many species, reflecting an involvement of dynamic PFC circuit [61] in which the PFC and hippocampus might communicate when PFC processes activity patterns formed in hippocampus during previous learning experience [62, 63].

Although both hippocampus and PFC are required for spatial learning, the hippocampus plays a vital role in rodent [64], while the PFC is more important in primates. These data suggest differentiated cognitive functions of the hippocampus and PFC between rodents and primates in working memory [65].

## 5. Oscillations Coordination and Molecular Regulation in the Hip-PFC Circuit

Oscillations across the frequency spectrum are evident and theorized to be relying on the strength and kinetics of excitatory and inhibitory synaptic interactions [66]. When large groups of neurons synchronize their electrical activity in a periodic manner, brain oscillations emerge in local field potential (LFP). Electrophysiological oscillations provide a mechanism of long-range interactions [67] and link hippocampus-PFC structural connectivity to PFC rhythmic electrical dynamics and memory performance. Oscillation of neuronal network activity in rodent includes theta (5–10 Hz), gamma (25–140 Hz), and sharp wave-associated ripple (150–300 Hz) [68]. Spindle (7–14 Hz) is a term used to describe cortical network activity [69].

### 5.1. Theta and Gamma Oscillations

Higher order cognitive processes such as working memory are strongly associated with theta and gamma oscillations in the PFC [70, 71]. The theta rhythm is an oscillatory pattern in EEG signals recorded either from inside the brain or from electrodes glued to the scalp. With localized gamma bursts transiently occurring at different mPFC locations, the theta rhythms cooccur with gamma oscillations in hippocampus and modulate mPFC gamma power [72, 73]. In the hippocampus, slow and fast gamma oscillations have been found to generate in different phases of theta preferentially [72, 74]. The hippocampal-mPFC coherence increases with slow gamma oscillation, suggesting that slow gamma oscillations contribute to coordinate interactions between hippocampus and mPFC. Consider the important role of mPFC in attentional selection, and fast gamma oscillations in the hippocampus are consistent with fast gamma oscillations in the medial entorhinal cortex, an area that transmits information to the hippocampus about the current environment [75]; it is likely that fast gamma oscillations may selectively activate neurons that code information related to attended stimuli.

Hippocampal LFP and mPFC spikes are phase-locked at a range of time shifts. Most mPFC spikes followed hippocampal theta instead of leading it suggesting an interaction of hippocampus and mPFC directionality [76]. Although mPFC theta oscillations were synchronized to both dorsal and ventral hippocampal theta, recent research indicates that Hip-PFC synchrony may depend primarily on activity in the vHip [72].

Study on gamma oscillation modulation in the mPFC uncovered that during sleep spindles the cortex is functionally disconnected with its hippocampal inputs which is presumably modulated by the strong recruitment of inhibitory interneurons [69]. The communication between local GABAergic fast-spiking and regular-spiking interneuron contributes to the transient formation of cell assemblies [77] associated with gamma oscillations [78]. Cortical theta and gamma rhythms depend on perisomatic inhibition of pyramidal neurons from hippocampal basket cells expressing cholecystokinin (CCK) and PV, respectively [79, 80]. Although the activity of multiple interneuron subtypes is linked with both theta and gamma rhythms in the hippocampus, the firing CCK cells are most strongly associated with theta oscillation [81]. In comparison, the firing PV cells show stronger association with gamma oscillation [82, 83]. It was suggested that a significant change of CCK- and PV-inhibition onto PFC pyramidal neurons may underlie working memory impairments and cortical oscillation loss [79].

### 5.2. Theta and Ripple Association

Hippocampal sharp waves (SPW) are associated with ripple pattern [84, 85] and are named as Sharp-Waves-Ripples (SPWRs). The spatial memories in rodents are formed during theta and gamma activity in the local EEG, while memory consolidation was proposed to be dependent on offline replay of previously stored information during SPW ripple association [86]. The shift from aroused to nonaroused state, such as from theta waves during walking to SPWRs during immobility, is modulated by the oscillation of hippocampus and PFC [87].

Hippocampal SPWRs are well placed to emphasize location of the animals. During the SPWRs, place cell firing is stronger when animal is located inside the cell's place-field as compared to outside. There is a nonlinear increase of the location-specific SPWRs firing rate as compared with that during theta periods [88], which promotes plasticity beyond that expected in areas in which SPWRs did not occur, enabling SPWRs to highlight locations of behavioral importance.

Switching between hippocampal theta and SPWRs may be reliant upon systemic administration of acetylcholine (ACh) [89], GABA [90], and norepinephrine [91]. The synaptic GABA release may more readily reach extrasynaptic GABA_B_ receptors upon blockade of GABA_A_ receptors by GABA_A_ receptor antagonist, bicuculline [90, 92]. A recent study shows that activation of GABA_B_ receptors promotes the transition from theta-gamma to SPWRs working mode in hippocampus [90] suggesting a critical role of GABA_B_R in SPWRs. It has also been shown that bicuculline augmented GABA_B_ receptor mediated inhibitory postsynaptic potentials (IPSCs) leading to SPWRs reduction [93, 94]. Thus the role of GABA_B_R in regulation of SPWRs remains to be elucidated.

### 5.3. SPWRs and Spindles

Behavioral study shows that PFC and hippocampus exhibit time-related correlation during the slow-wave sleep (SWS) [56]. In the hippocampus, SWS is marked by high-frequency network oscillations (~200 Hz ripples), whereas neocortical SWS activity is organized into low-frequency delta and spindle oscillations [56]. The slow oscillations of the neocortex are coordinated with spontaneous oscillatory activities in neocortex, entorhinal cortex, subiculum, and hippocampus [95]. SPWRs directly affect neocortical activity, especially in mPFC area that accepts monosynaptic fibers from subiculum and vHip. Thus, hippocampal SPWRs may be vital for transporting memory from hippocampus to PFC [96, 97].

During light SWS, the thalamocortical network oscillates falling in a waxing-and-waning pattern at about 7 to 14 Hz and lasting for 500 ms to 3 s, called spindles [98]. Both SPWRs and spindles have been manifested to be strongly involved in memory consolidation [84, 99]. SPWRs-spindle episodes were suggested to constitute a crucial mechanism of Hip-PFC information transfer during SWS [69] short-term hippocampal memory is transported to neocortex as long-term memory.

Using multisite neuronal recordings in mPFC, it has been shown that oscillatory responses of cortical cells differ due to different cell types and cortical layers during sleep spindles [69]. Superficial neurons are more tonically recruited and phase-locked during spindle episodes. In the deep layers of mPFC, with which most of the hippocampal fibers make contacts, pyramidal cells response was coupled to SPWRs, but not spindles [69]. Furthermore, in a given layer, interneurons are thought to play a crucial role in shaping pyramidal reaction in the mPFC in both firing rate and phase, possibly by regulating different neuromodulators, such as dopamine [100, 101].

## 6. The Hip-PFC Loop and Neuronal Disorders

Patients suffering from psychiatric and neurodegenerative diseases show cognitive impairment and emotional dysregulation as well as abnormalities of morphological, electrophysiological, and molecular properties in the Hip-PFC pathway. It is likely that disruption of the Hip-PFC functional connectivity might be a shared element of pathophysiology in the above disorders. We will discuss stress, depression, autism, and Alzheimer's disease in this review.

### 6.1. Stress and Depression

Posttraumatic stress disorder (PTSD) patients usually show abnormally small hippocampus, amygdala [102]. Neuroimaging data suggest that PTSD patients consistently display aberrant activity within the hippocampus and mPFC during fear-relevant tasks [103–105]. Pathological states of memory encountered in stress-related disorders are primarily associated with dysfunction of Hip-PFC interaction. Neuronal plasticity in the rodent, as assessed by LTP in projections from the subiculum to the mPFC, is blocked by prior stress, an effect that may be cardinal to some facets of psychiatric disorders [106].

Glucocorticoid (GR) and mineralocorticoid receptors (MR) not only display a different affinity for corticosterone, but also are heavily expressed in hippocampus, mPFC, and amygdala [107–109]. Concurrent enhancement of corticosterone after stress in dorsal hippocampus and mPFC causes a shift of memory retrieval pattern from dorsal hippocampus (nonstress condition) to mPFC, indicating that corticosterone is critically involved in mediating the deleterious effects of stress on cognitive functions involving the Hip-mPFC interaction [110].

Major depression is a mental disorder characterized by a pervasive and persistent low mood that is accompanied by low self-esteem and a loss of interest or pleasure in normally enjoyable activities [111, 112]. Depression is associated with altered morphology, anatomy, and pathophysiology in hippocampus and PFC [113, 114]. Depression patients often suffer from rumination and persistent thoughts associated with decreased hippocampal volume [115], disturbed activation of the mPFC [116], and disconnection of the cognitive control network involving the hippocampus and PFC [117]. Meanwhile, functional magnetic resonance imaging (fMRI) analyses revealed reduced Hip-PFC connectivity [117]. More recent study shows that attenuated theta phase coupling and theta-gamma cross frequency coupling of LPFs in the depression state may reflect impaired synaptic plasticity in Hip-PFC pathway [118].

The mature neuropeptide Y (NPY) is a 36-amino acid peptide, which is widely distributed in the nervous system [119, 120] and is classified into two types of* NPY* mRNA species: a “long” and a “short” variant [121]. Both functional analysis and genetic studies have shown that NPY is a crucial modulator of mental and emotional resilience [122, 123], including depression. The hippocampus and PFC have lower levels of both the “long” and the “short”* NPY* mRNAs, while only the “short”* NPY* mRNA was demonstrated to be downregulated in depression-like states in rat [124].

### 6.2. Autism Spectrum Disorder

Autism spectrum disorder (ASD) is a series of neurodevelopmental disorders defined by core deficits in repetitive behaviors and restrictive interests [125].

More recent results suggest that impaired glutamate N-methyl-D-aspartate receptor (NMDAR) dysfunction in the PL mPFC and hippocampus may contribute to impaired synaptic response associated with psychiatric illness [126]. Social withdrawal may be more closely aligned with NMDAR dysfunction in the hippocampus, while social intrusiveness may be more closely aligned with NMDAR dysfunction in the PL mPFC [126]. In addition to altered NMDAR function, both GABA_A_ [127] and GABA_B_ [128] receptors levels are significantly decreased, resulting in the heterogeneous disruption of excitation/inhibition (E/I) balance across the cortex [129], in postmortem brain samples from subjects with ASD. As glutamate and GABA receptors comprise the majority of ligand-gated ion channels in the CNS, these findings suggest that a disruption of E/I balance may contribute to the pathogenesis of ASD [129].

Neuroimaging studies have suggested that cortical interconnectivity is dysfunctional in ASD [130]. An aberrant increase in baseline spectral activity of gamma band and stimulus related nonphase-locked activity were manifested in ASD [131, 132]. Phase-locked activity is decreased in ASD individuals in comparison with age-matched controls. Thus, the study of circuit dysfunction in ASD, together with data obtained in other neuronal disorders, may charter the common cellular property of connectivity seen in multiple disorders and specific circuit abnormalities.

### 6.3. Alzheimer's Disease (AD)

AD is a progressive neurodegenerative disorder that causes deterioration of memory, judgment, and reasoning in the elderly. AD brain is characterized by accumulation of extracellular insoluble beta amyloid (A*β*) plaques and intracellular neurofibrillary tangles (NFTs) and selective synaptic and neuronal loss [133].

Both A*β* and NFTs impair hippocampal and cortical function [134]. The cortex functions as the gateway of neural projection between hippocampus and the rest of the brain, including PFC [135, 136]. Low frequency blood oxygenation level-dependent (BOLD) correlations linked with the Hip-PFC circuit reveal a structural and functional connectivity in neuronal disorders, such as Alzheimer's disease [137]. These functional connectivity relationships may provide a useful translatable probe of the hippocampal-PFC system for the further study of rodent models of disease and potential treatments. Strikingly, when functional connectivity between the right hippocampus and mPFC was disrupted in AD patients, the connectivity between the left hippocampus and right lateral PFC was increased [136]. Similarly, increased activity in the right dorsal lateral PFC [138, 139] and enhanced functional connectivity within the prefrontal regions [140] and/or between the PFC and other regions [139] were observed during memory tasks in AD patients, implicating that AD patients may be able to use excess neural projection in PFC to compensate impaired cognitive function [136].

It has been shown that reduced Nav1.1 levels of PV cell underlie abnormal memory and gamma oscillation in the cerebral cortex of AD mouse model and AD patient [141]. Restoring Nav1.1 levels enhanced inhibitory synaptic activity and gamma rhythms and decreased hypersynchrony, premature mortality, and memory deficits [141, 142]. Neurodegeneration and cognitive impairment in AD shift the EEG source-based spectral power and functional connectivity within the default mode network [143], characterized by consistent activation during a resting-state condition. These results implicate disrupted oscillatory activity as a potential neural marker of AD.

## 7. Closing Remark

Molecular biology, anatomy, electrophysiology, and fMRI analysis in rodent and human strongly implicate that the Hip-PFC circuit plays an important role in fundamental cognitive processes, emotion regulation, and memory consolidation. Disruption in the Hip-PFC pathway, anatomically or functionally, might be a common element of pathogenesis in neuronal disorders and therefore underlies the curious overlap of symptoms among these otherwise disparate diseased conditions. Thus, study of the physiology and pathophysiology within the Hip-PFC circuit may be a key for understanding these highly debilitating and prevalent psychiatric and neurodegenerative disorders.
